# Sizing and identification of nanoparticles by a tapered fiber†

**DOI:** 10.1039/c8ra06454g

**Published:** 2018-09-24

**Authors:** Huiling Pan, Weina Zhang, Hongxiang Lei

**Affiliations:** State Key Laboratory of Optoelectronic Materials and Technologies, School of Materials Science and Engineering, Sun Yat-Sen University Guangzhou 510275 China leihx@mail.sysu.edu.cn

## Abstract

There is a strong desire for sizing and identification of nanoparticles in fields of advanced nanotechnology and environmental protection. Although existing approaches can size the nanoparticles, or identify nanoparticles with different refractive indexes, a fast and simple method that combines the two functions still remains challenges. Here, we propose a versatile optical method to size and identify nanoparticles using an optical tapered fiber. By detecting reflection signals in real time, 400–600 nm SiO_2_ nanoparticles can be sized and 500 nm SiO_2_, PMMA, PS nanoparticles can be identified. This method requires only an optical tapered fiber, avoiding the use of elaborate nanostructures and making the device highly autonomous, flexible and compact. The demonstrated method provides a potentially powerful tool for biosensing, such as identification of nano-contaminant particles and biological pathogens.

## Abstract 2

Nanoparticles with different sizes or with different refractive index can be distinguished using an optical tapered fiber.

## Graphical abstract

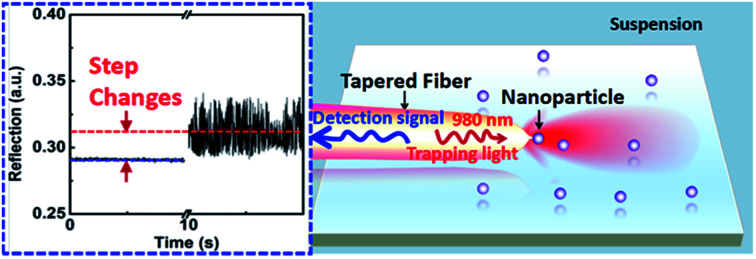

## Introduction

The sizing and identification of nanoparticles are becoming increasingly important for applications in various fields, such as environmental protection, disease diagnosis and treatment, as well as scientific research applications.^1–3^ For example, many of the body's physical diseases, including allergies and lung cancer, are caused by the inhalation of tiny particles, as well as particulates emitted from automobiles and industries, which also cause environmental pollution.^4–6^ Therefore, more attention should be paid to nanoparticles, including sizing and identification. There are many ways to size nanoparticles today, such as by using a scanning mobility particle sizer,^7^ laser particle analyzer^8^ and Scanning Electron Microscopy.^9^ Compared to the traditional methods, recently developed optical sizing techniques show great superiority in time response, low cost, and non-invasiveness in sizing particles.^10,11^ Especially for the techniques of microcavity sensing,^12–14^ interferometric scattering and photothermal microscopy,^15–17^ the detection limit is down to single nanoparticles. Among these methods, the microcavity sensing pursuits high *Q* factors up to 10^8^ and small mode volumes to enable significant enhancement of light–matter interactions; it can detect the nanoparticles as low as 30 nm, but it needs elaborate cavity system and a tunable laser source; the interferometric scattering can detect individual proteins with size less than 60 kDa *via* the interference of the light created by Rayleigh scattering and the reflection of the incident laser beam, and the photothermal microscopy can detect and count 20 nm gold nanoparticles, but both require carefully designed light path, a highly stable light source and extra imaging systems. By contrast, optical fiber-based sizing technique attracts much attention, because it is cavity-free, real-time and portable.^18–20^ The technique relies mainly on the nanofiber pairs or nanofiber-array structure with a strong evanescent field. The modified nanoparticles with positive charge could attached to the negatively charged nanofiber in an aqueous environment, and thus an ultrasensitive sizing sensor can be obtained by detecting the transmitted power of the fiber. In the methods, the structure is generally disposable and the thin nanofiber also may lead to weaker mechanical properties. In addition to the sizing sensing of nanoparticles, refractive index sensing is also crucially important. Typical refractive index sensing^21–23^ relies on local surface plasmon resonance (LSPR) to achieve high accuracy and sensitivity. The sensitivity can reach to 3.2 × 10^−5^ RIU, but when applied to nanoparticles, the range of detectable refractive indices is about 1.33–1.40, which cannot meet the actual requirements.

These above difficulties make us have a strong demand to explore a fast, simple, sensitive and stable technique can both size nanoparticles and identify particles with different refractive index. Since the recently reported fiber optical tweezers is a good platform for stable trapping and precise manipulation of nanoparticles^24–26^ and for real-time detecting and analysis of nanoparticles or cells,^27,28^ all these motivate us consider to use fiber optical tweezers to size and identify nanoparticles. Therefore, in the work, we present a simple, low-cost and high-sensitivity system for quickly sizing and identifying nanoparticles in an aqueous environment by a tapered fiber (TF). After being stably trapped under an action of optical force, different nanoparticles can be identified by monitoring the step changes of their reflecting signals. This method requires only an optical tapered fiber that can be used to both trap and monitor different nanoparticles, avoiding the use of elaborate nanostructures and making the device highly autonomous, flexible and compact.

## Experimental schematic

**Fig. 1:**
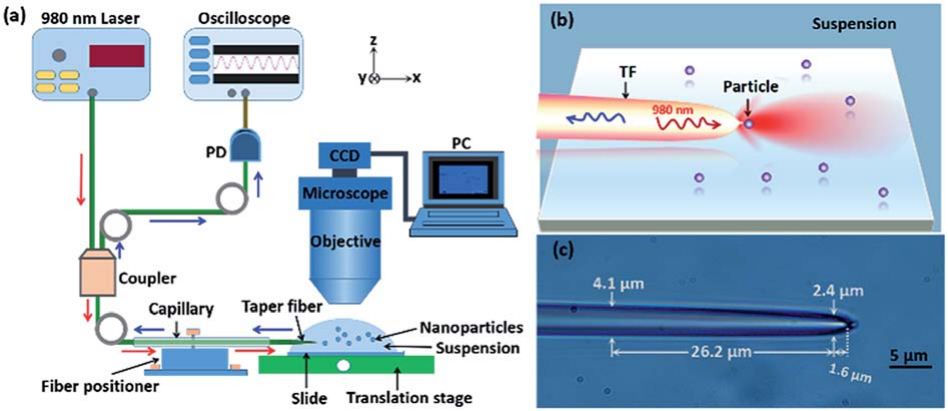
(a) Schematic of experimental setup. The red and blue arrow indicate the 980 nm light input into the TF and the reflecting signals delivered to the oscilloscope, respectively. (b) Schematic of sizing and identifying a single nanoparticle. The wavy red and blue arrows indicate the input light and reflecting signal, respectively. (c) Optical microscopic image of the TF used in the experiment.

Fig. 1(a) shows the schematic of experimental setup. A tapered fiber (TF), fabricated by drawing a commercial single-mode optical fiber through a flame-heating method,^28^ was sheathed partly by a glass capillary and then mounted on the tunable six-axis fiber positioners (Kohzu Precision Co., Ltd.). The tip of the fiber was immersed in a solution with nanoparticles. The other end of the TF was connected to a 1 × 2 fiber coupler. A 980 nm laser beam, which is weakly absorbed by most nanoparticles and biological samples (see the detailed description in Fig. S1 in ESI†). The 980 nm laser was directly injected into one arm of the coupler, while the other arm of the coupler was connected to an oscilloscope through an InGaAs-based photoelectric detector (Thorlabs DET01CFC, bandpass: 790–1200 nm) in order to detect the reflection signal in real time. As a result, in this configuration, both trapping and detection were performed with only a tapered fiber. To observe and capture the experimental process, a computer interfaced microscope with a CCD camera was used. The mechanism of trapping and identifying nanoparticles is shown in Fig. 1(b). Because the 980 nm laser beam is strongly focused at the tip of the fiber, thus the nanoparticles near the tip will be stably trapped under an action of optical force. At this time, the trapped nanoparticle will reflect a portion of the field energy to the environment. Because the tip of the TF also acted as a high NA objective, the reflection signal of the trapped nanoparticle can be detected in real time. By monitoring the step changes in the reflecting signals, sizing and identification of a single nanoparticle can be demonstrated experimentally. Based on the method, for some small nanoparticles those are difficult to be distinguished directly with an optical microscope, we can obtain the size and refractive index of the nanoparticles by observing the feedback reflecting signal from the oscilloscope. The TF used in the experiments is shown in Fig. 1(c). The diameter is gradually reduced from 4.1 to 2.4 μm over a length of 26.2 μm and then the TF is rapidly ended up with a taper in the 1.6 μm range. It has a good mechanical property and can be used repeatedly (see the fabrication of TF in details in Methods). It should be pointed out, the structure parameters of fiber, including the effective length of the fused zone (2.0 mm), diameter (2.4 μm) and taper angle (70°), are very important for trapping nanoparticles. Too long or too short length of the fused zone will induce a strong transmission loss of fiber. Inappropriate length of diameter and taper angle will induce an unstable or failed trapping. As examples, Fig. S2 and S3 in ESI† show the simulated optical field of a trapped 500 nm SiO_2_ particle by the tapered fiber with different diameter and taper angle, which further proves that the structure parameters of fiber we choose is suitable for trapping the nanoparticles. As an experimental example, Fig. S4 in ESI† shows that, a 500 nm SiO_2_ particle can also be trapped using a taper fiber with diameter of 2.4 μm and taper angle is 45°, which is consistent with the simulated conclusion in Fig. S3.† Here, it should also be pointed out that, the multi-mode fiber (MMF) can not affect the trapping situation of the nanoparticles and then the reflected signals (see Fig. S5 in the ESI†).

## Results and discussion

**Fig. 2:**
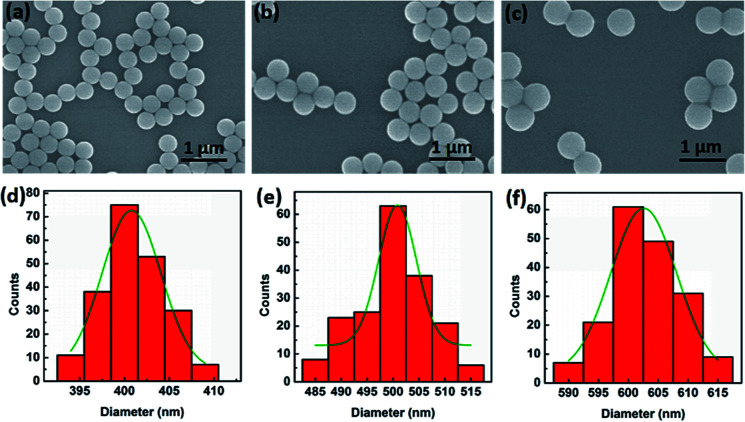
Characterization of the SiO_2_ nanoparticles. (a)–(c) SEM images of 400, 500 and 600 nm SiO_2_ particles, respectively. (d)–(f) Diameter distribution of 400, 500 and 600 nm SiO_2_ particles, respectively. The green lines are the corresponding Gaussian fitting.

In our experiment for sizing nanoparticles, we used five different suspensions of silica (SiO_2_) monodisperse nanoparticles with diameters of 400, 450, 500, 550, 600 nm, respectively. To clearly characterize the morphologies of nanoparticles, as an example, Fig. 2(a)–(c) present scanning electron microscope (SEM) images of SiO_2_ nanoparticles with the diameter of 400, 500, and 600 nm, respectively. We can see that the size of SiO_2_ nanoparticles are uniform. Fig. 2(d)–(f) present their diameter distribution and the corresponding Gaussian fitting, respectively. The standard deviation of particle size is less than 10 nm.

**Fig. 3:**
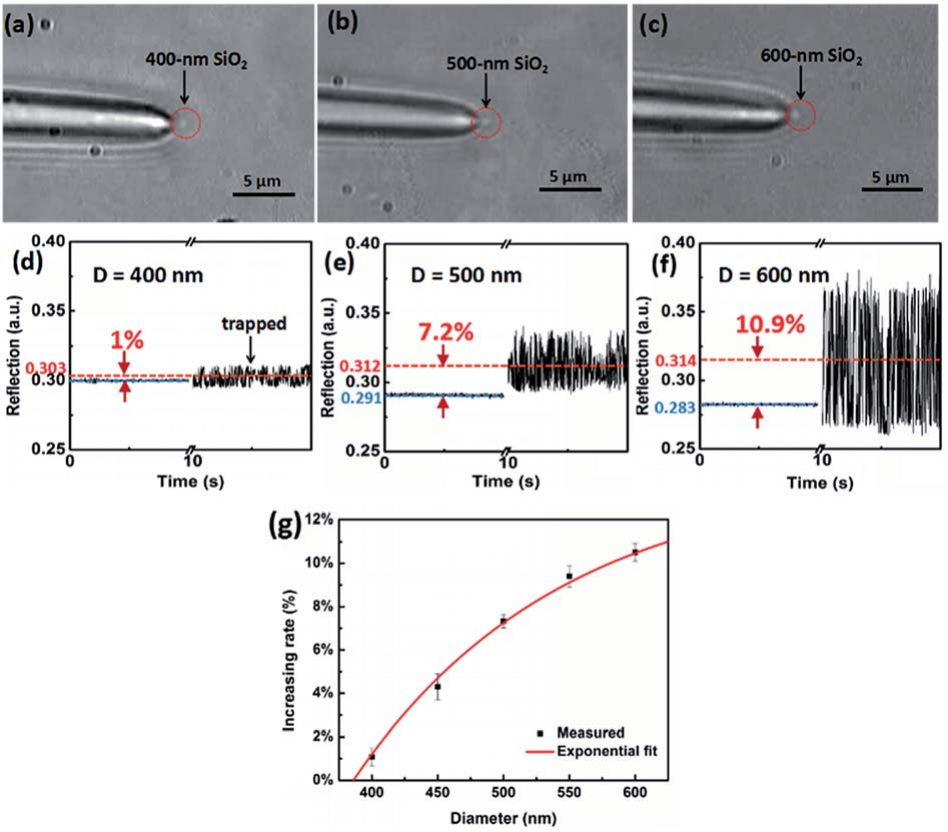
Sizing of a single SiO_2_ nanoparticle. (a)–(c) The optical microscope images of trapped SiO_2_ nanoparticle with diameters of 400, 500 and 600 nm, respectively. (d)–(f) Real-time capture of reflection signals for the trapped SiO_2_ particle with diameters of 400, 500 and 600 nm, respectively. (g) Signal increasing rate as a function of particle diameter.

Optical power of the trapping light from the tip of the TF was set to 12 mW. Once the light was launched into the tip, a single SiO_2_ nanoparticle near the tip can be stably trapped under an action of optical force. Moreover, by monitoring the reflecting signal of the trapped nanoparticle, an obvious step change can be observed in the oscilloscope. In addition to observing the diameters of the nanoparticle directly, the sizes of the nanoparticle can also be obtained and analyzed through the real-time reflection signals. For the different size of nanoparticles, the intensities of reflecting signal and thus the height of step change are also different. For examples, Fig. 3(a)–(c) show three optical microscope images of a single 400, 500, 600 nm SiO_2_ nanoparticles trapped stably by the TF, respectively. Compared with the bare fiber in Fig. 1(c), the captured nanoparticle is at the end of the fiber and along the fiber axis. Fig. 3(d)–(f) show the corresponding reflecting signals monitored in the oscilloscope, respectively. The vibration of trapping a nanoparticle was observed because of the strong Brownian motion, indicating that the nanoparticle was trapped rather than stick on the surface of the TF. Moreover, when the nanoparticle was trapped, the signal intensity was higher than that of uncaptured signal and the step changes of the signal can be used to size and identify the nanoparticles. In our experiment, the mean uncaptured nanoparticle signal value (indicated with blue lines) and captured nanoparticle signal value (indicated with red lines) are set to *I*_1_ and *I*_2_, respectively. The signal increasing rate (*I*_*i*_) was obtained from *I*_*i*_ = (*I*_2_ − *I*_1_)/*I*_1_, and the measured *I*_*i*_ values of particles with the diameter of 400, 450, 500, 550, 600 nm are 1%, 4.3%, 7.25%, 9.2%, 10.9%, respectively. Fig. 3(g) shows the increasing rate for single captured nanoparticles with different diameters. The increasing rate *I*_*i*_ is increased exponentially with the diameter *D*. It is mainly because that the increasing diameter can induce much more reflection light. A fitted exponential expression between *I*_*i*_ and *D* is given by

1 *I*_*i*_ = *A*_1_ exp(−*D*/*t*_1_) + *I*_*i*0_

where the fitted values of *A*_1_, *t*_1_, and *I*_*i*0_ are −1.47, 168.16 nm and 0.147, respectively. This expression intuitively provides the relationship between the particle diameter and the increasing rate of the reflection signal. Thus, we can directly obtain the diameter of the SiO_2_ particles of unknown size with this expression. In our experiment, the fitted exponential expression can be used for the sizing of SiO_2_ particles of 400–600 nm, which is difficult to be directly measured under optical microscope. The sensitivity *S* of the system is defined as *S* = |Δ*I*/Δ*d*|, where Δ*I* is the change of the step changes of the signals and Δ*d* is the corresponding size difference of two particles. From Fig. 3(g), we have calculated that the sensitivity is about 1.4 × 10^−4^/nm. Since the resolution of the oscilloscope used is 1 × 10^−3^, therefore, the detecting precision for different size is about 7 nm in this system.

**Fig. 4:**
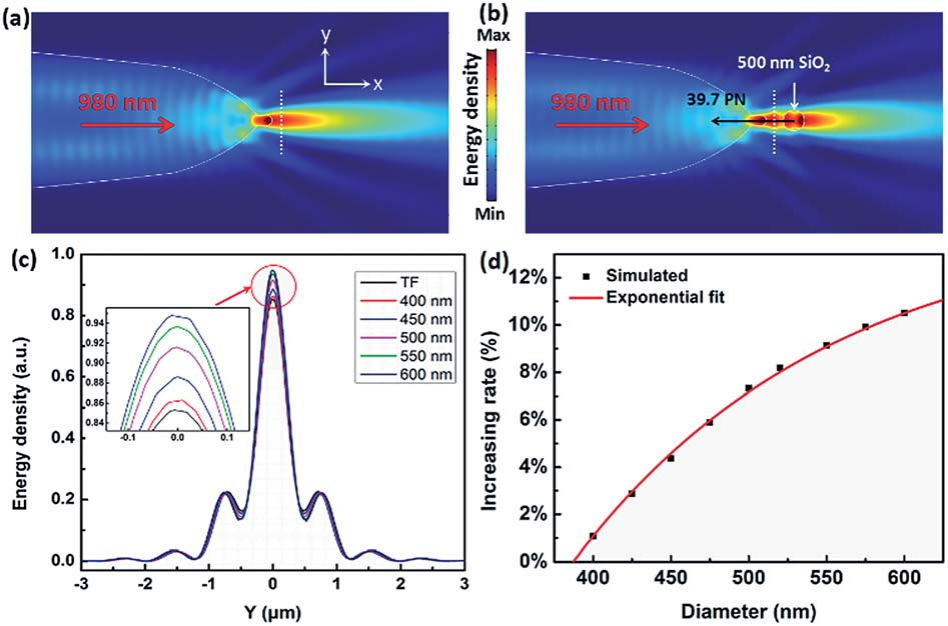
Simulation and calculation results. Simulated optical energy density distributions of (a) a single TF and (b) a 500 nm SiO_2_ particle trapped by the TF. (c) Energy density intensity in the direction of white line of (a and b) and other sizes of SiO_2_ particles. (d) Signal increasing rate as a function of diameter.

The above experimental results show that the TF can be used to trap and size SiO_2_ nanoparticles. In order to explain this phenomenon numerically, a finite-element method using COMSOL Multiphysics (see the Methods section for details) was applied to simulate the distribution of the light field and calculate the force acted on the trapped nanoparticles. In the simulation, the shape of the optical fiber was set to be the same as that of Fig. 1(c). The shape of the SiO_2_ nanoparticles was set to be a sphere, and the diameter was set to be the same as the actual size. The refractive indices of the TF, water, and SiO_2_ particle were set to be 1.44, 1.39, and 1.44, respectively. The optical power of 980 nm laser beam was set to be 1 W. The optical field distribution output from a single fiber is shown in Fig. 4(a). The output light is highly concentrated at the tip of the TF so that nanoparticles can be stably trapped. As an example, Fig. 4(b) shows the simulated optical field distribution of a trapped 500 nm SiO_2_ particle. In order to perform the trapping ability of the TF, the optical force (**F**_O_) exerted on the nanoparticle is calculated by integrating the time-independent Maxwell stress tensor 〈**T**_M_〉 on the surface of SiO_2_ particle according to ref. 292
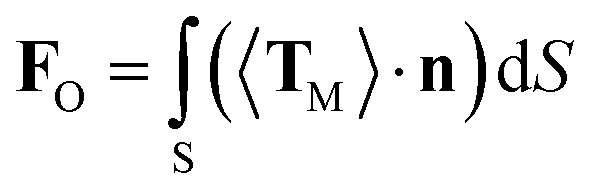
where the integration is performed through a closed *S* surface around the nanoparticle, **n** is the surface normal vector of *S*, and 〈**T**_M_〉 is the time-averaged Maxwell stress tensor, which is given by3
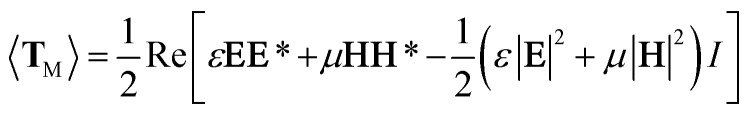
where *I* is the unit dyadic, and *ε* and *μ* are the dielectric constant and magnetic permeability of the surroundings, EE* and HH* stand for the outer product of the optical fields, respectively. Using this method, the optical force exerted on the nanoparticles can be calculated. As a result, the *F*_*x*_ in the *x* direction (along the fiber axis) of 400, 500, 600 nm SiO_2_ particles is −33.9, −39.7, −65.2 pN, respectively, indicating that the optical force exerted on the nanoparticle is optical gradient force, which increases with an increasing diameter of particle. These values are in good agreement with the reflection signal results described above. The optical force becomes significantly stronger as the nanoparticles become larger in a manner is proportional to the cubic power of size.^30^ This is because as the particle size increases, the Brownian motion is relatively weakened and it is more difficult for the particles to escape the capture of the fiber.

To theoretically analyze the relationship between the reflection signal and the size of the nanoparticles, we compare the difference between the reflection signal of the bare fiber and the captured particles with different diameter. Line scans were performed through the white dotted lines of Fig. 4(a) and (b), and the results show that all curves show similar shapes in Fig. 4(c). In order to facilitate observation and comparison of the reflected signals at different particle diameters, the inset of Fig. 4(c) shows an enlarged view of a selected area. The intensity of energy density decreases in order of 600, 550, 500, 450, 400 nm SiO_2_ nanoparticle, bare TF. We noticed that the highest value of energy density was also showing this trend. Because the reflection signal of captured nanoparticle contains the signal of bare fiber, in order to obtain the real reflection signal of nanoparticles, the signal of bare fiber need to be removed. Here, we set the energy density of the bare fiber to *R*_0_ and the energy density for trapping different sizes of SiO_2_ nanoparticles as *R*_*i*_ (*i* = 1, 2, 3…). Thus, the real reflection signal of nanoparticle is (*R*_*i*_ − *R*_0_) and the increasing rate *R*_r_ = (*R*_*i*_ − *R*_0_)/*R*_0_, which are similar to the parameters set by the experimental results above. Fig. 4(d) shows the increasing rate for trapping a single SiO_2_ nanoparticle with different diameters. The increasing rate *R*_r_ is increased exponentially with the diameter *D*, which is consistent with the previous experimental results. A fitted exponential expression between *R*_*i*_ and *D* is given by

4 *R*_r_ = *A*_2_ exp(−*D*/*t*_2_) + *R*_r0_

where the fitted values of *A*_2_, *t*_2_, and *R*_r0_ are −1.50, 165.58 nm and 0.145, respectively. The simulated exponential results agree shows a good agreement with the previous experimental results, where *A*_1_, *t*_1_, and *I*_*i*0_ are −1.47, 168.16 nm and 0.147, respectively. This also confirms that the signal transmitted by the captured nanoparticles to the fiber is reflection signal.

**Fig. 5:**
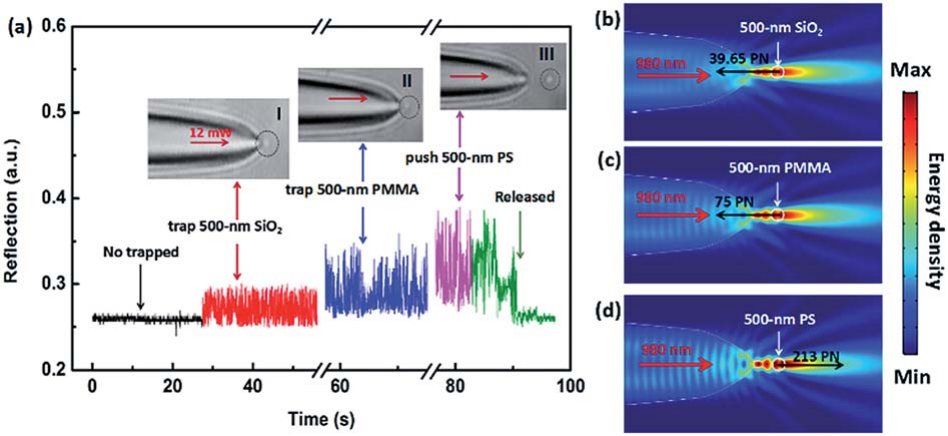
(a) Reflection signal from the trapping and pushing process in real time; inset I–III show the capture images of a 500 nm SiO_2_ and PMMA particles and the propel image of a 500 nm PS particle, respectively. (b)–(d) Simulated optical energy density distributions of a 500 nm SiO_2_, PMMA and PS nanoparticles manipulated by the TF, respectively.

In the above experiment, we utilized the step changes of the reflection signal to size the SiO_2_ nanoparticles. We consider that the size change of the particles will affect the reflection signal, and changes in the refractive index may also produce the similar result on the signal. In order to distinguish the nanoparticles with different refractive indices by the optical fiber, we used 500 nm SiO_2_, PMMA (polymethyl methacrylate) and PS (polystyrene) particles for experiments, respectively. The results show that the SiO_2_ nanoparticle and PMMA nanoparticle were trapped by the TF with an optical power of 12 mW, but the PS nanoparticle was pushed away. This is mainly due to the refractive index of PS nanoparticle is larger than the first two. In our experiment, the reflection signal of the manipulating process was monitored in real time. Fig. 5(a) shows the five real-time signal regions, which correspond to five manipulating process. Inset I–III show the trapping images of a 500 nm SiO_2_ and PMMA microsphere, and the propel image of a 500 nm PS particle, respectively. With the 500 nm SiO_2_ particle trapped (inset I), the intensity of the reflection signal was enhanced and the step changes were formed. Compared with 500 nm SiO_2_ particle, the 500 nm PMMA microsphere (inset II) has a stronger reflection signal intensity and step changes. Although the 500 nm PS particle was pushed away by the TF (inset III), it still has the strongest reflection signal among the three nanoparticles. Part 5 (green line) shows that the reflection signal returned to the original uncaptured state when the fiber was moved away from the trapped particles or the particles was pushed far away while the laser was still on.

To further explore the reasons for the formation of this phenomenon, we analyze the simulated light field distribution of the three particles. Fig. 5(b)–(d) show the simulated light field distribution of trapping a 500 nm SiO_2_, PMMA, and pushing a 500 nm PS particle, respectively. The parameters setting was the same as those of Fig. 4(a) and (b). The refractive indices of SiO_2_, PMMA, and PS were set to be 1.44, 1.49, and 1.58, respectively. When the incident light power of 980 nm laser was normalized to be 1 W, the calculated forces (*F*_*x*_) of 500 nm SiO_2_, PMMA, and PS particle were −39.65, −75, 213 pN, respectively, indicating that the optical forces exerted on the SiO_2_ and PMMA particles is the trapping forces, and the force applied on the PS is the driving force,^23^ which is consistent with the experimental results above. Additionally, according to the energy density distribution in the simulation diagram, it can be clearly seen that the reflected energy density of the PMMA particle is stronger than that of the SiO_2_ particle, and it is weaker than that of the PS particle, which is consistent with the above conclusion. The reason for the above phenomena can be attributed to the difference in the refractive indices of the three nanoparticles. The experimental results indicate that, for the same size of nanoparticles, higher refractive index will generate stronger reflecting signal. Moreover, for the nanoparticles with a high refractive index, their capture state will become the pushing state, which can be used to selectively trap nanoparticles and separate the nanoparticles.

Theoretically, our system can detect the optically transparent samples (for 980 nm wavelength) with refractive index larger than that of the surrounding liquid (1.33 in our work). They can be dielectric nanoparticles, semiconductor nanoparticles, biological samples and so on. For dielectric nanoparticles, in addition to SiO_2_ nanoparticles, other dielectric nanoparticles, such as PS and PMMA particles can be also sized. For example, Fig. S6 in ESI† shows the optical microscope images and real-time capture signals of a single PMMA particle with diameters of 500, 700 nm and 1 μm, respectively. For large-size semiconductor optical transparent materials, such as TiO_2_ nanoparticle with hundreds of nanometers in diameter (the refractive index: 2.49), it can be also detected. For example, Fig. S7 in the ESI† shows that an 800 nm TiO_2_ particle can be trapped by the tapered fiber with diameter of 2.4 μm and the taper angle of 45°, and the corresponding reflected signals can be also detected. Therefore, the refractive index range we can detect in the experiment is 1.44–2.49. In addition, this method is also applicable to biological samples with optically transparent properties, for example, *Staphylococcus aureus* (*S. aureus*) with similar morphology with nanoparticles. *S. aureus* is an important pathogen of human beings, closely related to food safety issues, and can cause many serious infections. Therefore, the detection of *S. aureus* is of great significance. Fig. S8 in ESI† shows the optical microscope images and real-time capture signals of a single *S. aureus* with different diameters. Additionally, for the optically transparent samples (for 980 nm wavelength) with excitation properties, such as down-conversion luminescent material, the excitation light and the emission light are generally UV-light or visible light and the short wavelength light can't be detected by the photoelectric detector in our system (bandpass: 790–1200 nm), so the excitation spectral properties can't affect the reflected signals.

Based on the above, the proposed tapered fiber can be used to trap and distinguish 400–600 nm nanoparticles those are almost blurry in the optical microscope, which is of great significance to the physical sciences. Moreover, due to the bare fiber's capture limit, the use of a single, unmodified tapered fiber to trap particles as small as 400 nm is also a major technological advancement in this experiment. This optical method is simple and fast, does not require special treatment of the sample, and does not require complicated equipment. Only a tapered fiber and a laser source are sufficient. Furthermore, it can be also used to identify other materials, such as cells or bacteria. Here, cell or bacteria viability was not affected by the incident laser at the low optical power. The fiber can also be manipulated in living bodies, such as the plant,^31^ which will be of great significance for the bioscience. Therefore, in future applications, this method not only can be used to identify pollution particles in the environment, but also can identify pathogens that are harmful to the human body. In addition, because fibers with different shapes can trap particle of different sizes and refractive index ranges, and due to the reusability of optical fibers, the preparation of multiple optical fibers allows us to broaden the species of detection object. If the end of the fiber were decorated with a microsphere to form a photonic nanojet, it can overcome the diffraction limit and be further used to detect and identify sub-100 nm nanoparticles, which will have an important impact on the fields of biology, physics, environment, materials and so on.

## Conclusion

In summary, an optical method was performed for trapping and distinguishing nanoparticles using a tapered fiber. By launching a 980 nm laser beam with an optical power of 12 mW into the fiber probe, the nearby nanoparticles can be trapped or pushed under an action of optical force and their reflection signals can be detected in real time. Based on this method, different sizes of SiO_2_ particles between 400–600 nm can be discerned with a measurement resolution of 50 nm in our experiment, and 500 nm SiO_2_, PMMA, PS particles with different refractive index can also be distinguished. This flexible and simple particle capture and analysis strategy provides a new approach for nanoparticle detection, sensing and real-time analysis. Moreover, this optical method also provides new opportunities for the discrimination of contaminant particles and biological pathogens.

## Methods

### Fabrication of the TF

The tapered fiber was fabricated by a flame-heating technique from a commercial single-mode optical fiber (connector type: FC/PC, core diameter: 9 μm, cladding diameter: 125 μm). A fiber stripper was used to strip the fiber's buffer layer and polymer jacket to produce a bare fiber with a length of 3 cm and a diameter of 125 μm. Before heating, the fibers were wrapped in glass capillaries to ensure fiber stability. When the naked optical fiber outside the capillary is heated by an alcohol lamp, the external flame was about 480 °C, and it was held for 20 s to reach its external melting point. The fiber was drawn at a speed of ∼2 mm s^−1^ with a heating zone about 3 mm, the fiber was gradually tapered off, its diameter was reduced from 125 to 5 μm, and the length is about 1.5 mm. Finally, the stretching speed was increased to ∼10 mm s ^−1^ until the fiber ends with a tapered tip.

### Preparation of the particle suspension

Commercially available monodisperse silica (SiO_2_), polymethyl methacrylate (PMMA) and polystyrene (PS) particles (Shanghai Huge Biotechnology Co., Ltd, Shanghai, China) have refractive indices of 1.44, 1.49, and 1.58, respectively. The SiO_2_ particles of different sizes were separately added with deionized water according to the same concentration, and then ultrasonically to make them uniform, the concentration was approximately 2.1 × 10^3^ particles per μl. SiO_2_, PMMA and PS particles were prepared in the same way. Then the particle solution was dropped onto the glass slide by a pipette for the following experiment.

### Simulation and calculations

Using COMSOL Multiphysics 5.3 with radio frequency modules (electromagnetic waves, frequency domain) and matching layer boundary conditions, the simulated optical field distribution was simulated by the finite element method. The incident light introduced into the fiber probe is set to a Gaussian beam with a wavelength of 980 nm. The refractive indices of the fiber probe, SiO_2_ nanoparticles, PMMA nanoparticles, PS nanoparticles, and water were set to 1.44, 1.44, 1.49, 1.58, and 1.33, respectively.

## Conflicts of interest

There are no conflicts to declare.

## Supplementary Material

RA-008-C8RA06454G-s001
