# Supplementary material for: Sizing and identification of nanoparticles by a tapered fiber
Source: RSC Adv. 2018 Sep 24;8(57):32916–21. doi: 10.1039/c8ra06454g (PMC9086333; doi:10.1039/c8ra06454g)
Supplement: RA-008-C8RA06454G-s001 [file RA-008-C8RA06454G-s001.pdf]

Electronic Supplementary Material (ESI) for RSC Advances.
This journal is © The Royal Society of Chemistry 2018

# Supplementary Information

## Sizing and identification of nanoparticles by a tapered fiber

Huiling Pan, Weina Zhang and Hongxiang Lei*

State Key Laboratory of Optoelectronic Materials and Technologies, School of Materials Science and Engineering, Sun Yat-Sen University, Guangzhou 510275, China

*E-mail: leihx@mail.sysu.edu.cn;

### 1. Simulated optical field distributions of a tapered fiber with different wavelengths of the laser.

In order to explore the impact on the reflection signal with different wavelength lasers, Figs. S1a–d show the simulated optical field of the tapered fiber with 671, 808, 980, 1064 nm wavelength lasers, respectively. Line scans were performed through the white dotted lines of Figs. S1a–d, and the results are shown in Fig. S1e. Fig. S1e shows that the full width at half maximum (FWHM) of the laser beam at the wavelength of 671, 808, 980, 1064 nm is 341, 426, 537, 578 nm, respectively. Therefore, the laser with the shorter wavelength can obtain the smaller focus spot and larger energy density, and thus the reflection signal will be stronger. Considering the short wavelength laser beam (i.e., visible light) can kill the biological cells and the long wavelength laser beam (i.e., far infrared light) can induce the unstable trapping because of the thermal effect, the researchers often choose to use near infrared light to trap and manipulate particles or biological samples. Therefore, for the potential biological and medical applications, 808 and 980 nm laser are more suitable. In our work, we use 980 nm laser to size and identify nanoparticles.

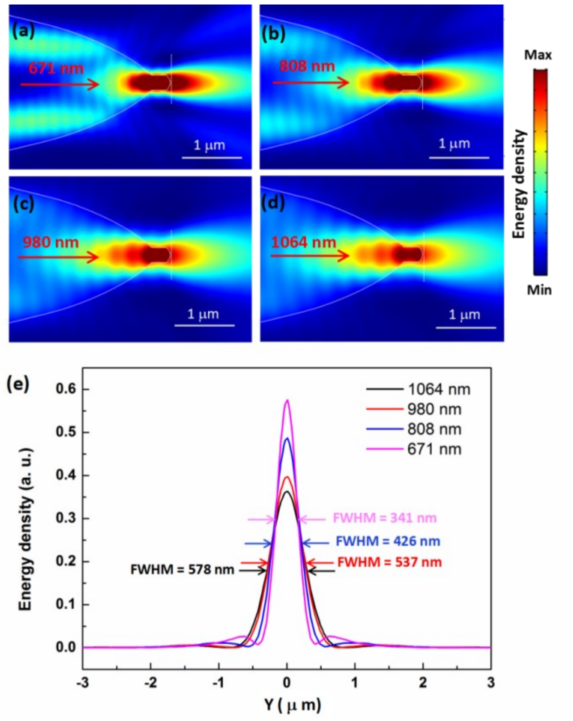


**Fig. S1 (a–d)** Simulated optical field distributions of the tapered fiber with different wavelength lasers. **(e)** Energy density distribution of the laser beams with different wavelengths.

**2. Simulated optical field distributions of trapping a 500 nm $SiO_2$ particle by a taper fiber with different diameters.**

Figs. S2a–d show the simulated optical field for the tapered fiber with diameters of 1.6, 2.4, 3.0 and 3.6 μm, respectively, and with the same length of the fused zone (2 mm in the paper) and taper angle (70° in the paper). The optical forces exerted on the $SiO_2$ particle by the four tapered fibers are −20.6, −39.7, 4.1, 51.9 pN, respectively. The simulation results show that, for the tapered fiber with the same length of the fused zone (2 mm) and taper angle (70°) in the paper, the $SiO_2$ particles will be pushed away when the diameter is over 3 μm, which further proves that the diameter we choose (2.4 μm) is suitable for trapping the nanoparticles.

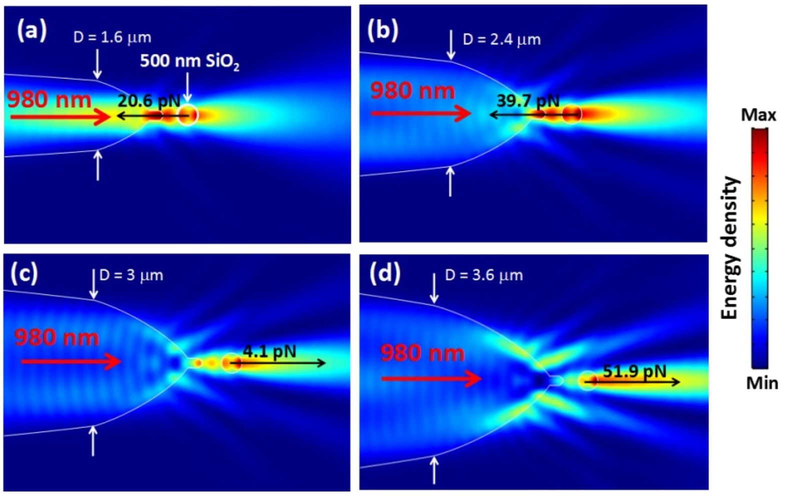


**Fig. S2 Simulation and calculation results. (a–d)** Simulated optical energy density distributions of a trapped 500 nm $SiO_2$ particle by a tapered fiber with the diameters of 1.6, 2.4, 3, 3.6 μm, respectively.

### 3. Simulated optical field distributions of trapping a 500 nm $SiO_2$ particle by a taper fiber with different taper angles.

Figs. S3a–e show the simulated optical field for a tapered fiber with taper angle of 35°, 50°, 70°, 75°, 80°, respectively, and with the same length of the fused zone (2 mm in the paper) and diameter (2.4 μm in the paper). The optical forces exerted on the $SiO_2$ particle by the five tapered fibers are –0.42, –31.2, -39.7, 3.7, 27.1 pN, respectively. For the tapered fiber with the same length of the fused zone (2 mm) and diameter (2.4 μm), it's suitable to trap the nanoparticles with the taper angle range of 35° –75°, which further proves that the taper angle we choose (70°) is suitable for trapping the nanoparticles.

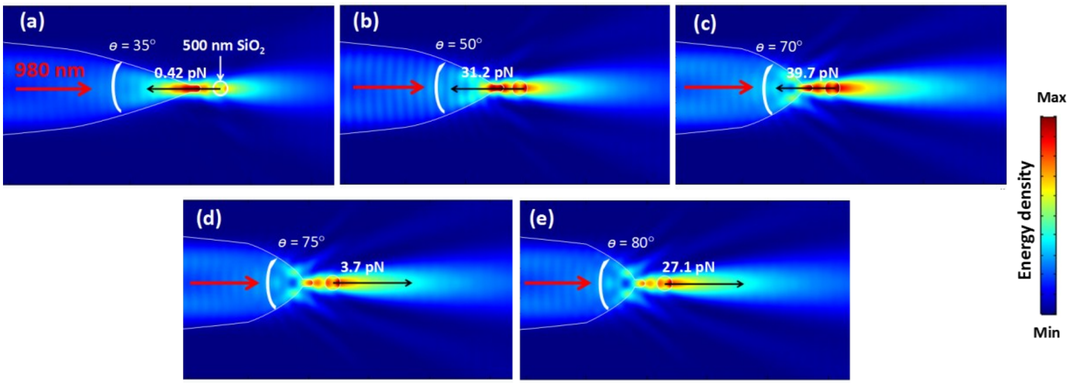


**Fig. S3 Simulation and calculation results. (a–e)** Simulated optical energy density distributions of a trapped 500 nm $SiO_2$ particle by a tapered fiber with taper angles of 35°, 50°, 70°, 75°, 80°, respectively.

**4. The optical microscope images of a trapped 500 nm $SiO_2$ particle by a tapered fiber with taper angle of 45° and diameter of 2.4 μm.**

As an experimental example, Figure S4 shows that, a 500 nm $SiO_2$ particle can also be trapped using a tapered fiber with diameter of 2.4 μm and taper angle of 45°, which is consistent with the above simulated conclusion in Fig. S3.

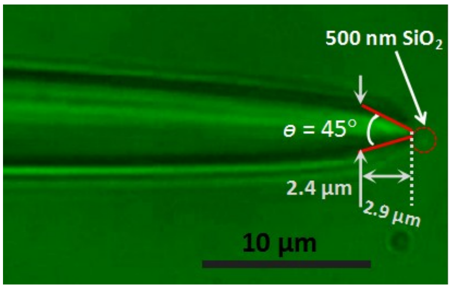


**Fig. S4** Optical microscope images of a tapered fiber trap 500 nm $SiO_2$ with the taper angle of 45° when the diameter is 2.4 μm.

**5. Simulated optical field distributions of a trapped 500 nm $SiO_2$ particle by a 4 μm-diameter multi-mode fiber (MMF) with taper angle of 40° and 70°.**

To prove the multi-mode fiber (MMF) can not affect the trapping situation of the nanoparticles and then the reflected signals, as an example, Fig. S5a, b shows the simulated optical field of a trapped 500 nm $SiO_2$ particle by a 4μm-diameter multi-mode fiber (MMF) with taper angle of 40° and 70°. The optical forces exerted on the particle are 10.1 and 52.8 pN, respectively, which will make the particle pushed away. Based on Fig. S2 and Fig. S5, we can deduce that, in order to stably capture the nanoparticles, the diameter must be less than 3~4 μm. In fact, when the multi-mode fiber (MMF) is pulled down to this size by the flame-heating method, it is no longer different from the single-mode fiber.

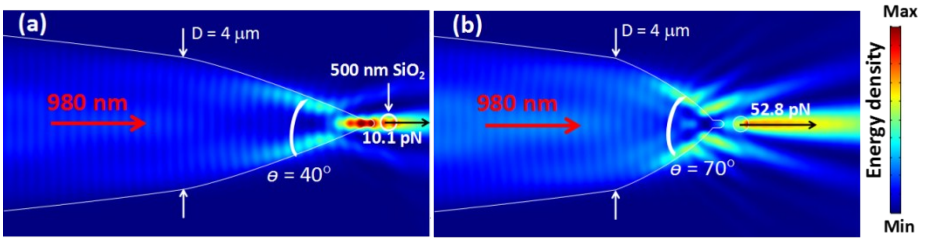


**Fig. S5 Simulation and calculation results.** Simulated optical energy density distributions of a trapped 500 nm $SiO_2$ particle by a 4 μm MMF with taper angles of 40° **(a)** and 70° **(b)**, respectively.

**6. The optical microscope images and reflection signal of trapping 500, 700 nm, 1 μm PMMA particle.**

Fig. S6 shows the optical microscope images and real-time capture signals of

trapped PMMA particle with diameters of 500, 700 nm and 1 μm, respectively. The results show that PMMA particles with different diameters can also be sized by the step changes of signal, and the amplitude of the step changes of signal increases with the increasing diameter of the PMMA particles, which is consistent with the conclusions in the paper.

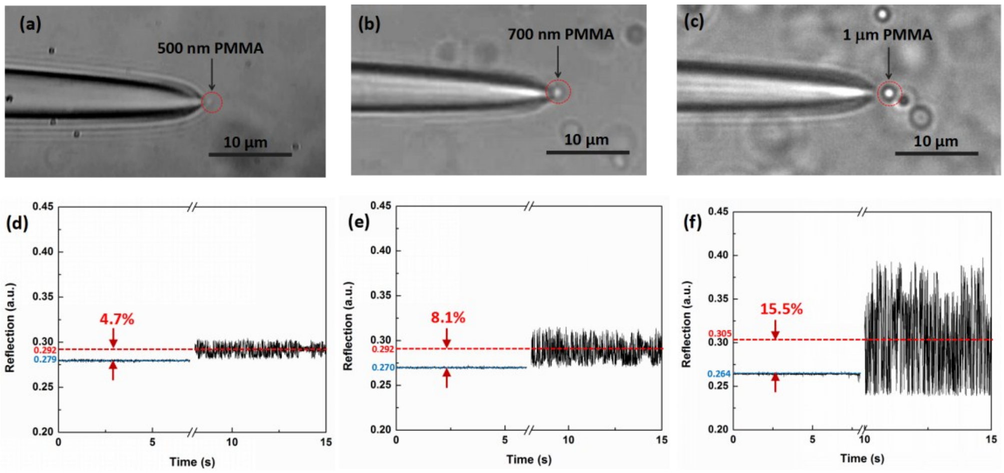


**Fig. S6 (a–c)** Optical microscope images of trapping 500, 700 nm, 1 μm PMMA particle, respectively. **(d–f)** Real-time capture of reflection signal for the trapped 500, 700 nm, 1 μm PMMA particle, respectively.

## 7. The optical microscope image and reflection signal of trapping 800 nm $TiO_2$ particle.

Fig. S7 shows that an 800 nm $TiO_2$ particle can be trapped by the tapered fiber with diameter of 2.4 μm and taper angle of 45°, and the corresponding reflected signals can be detected.

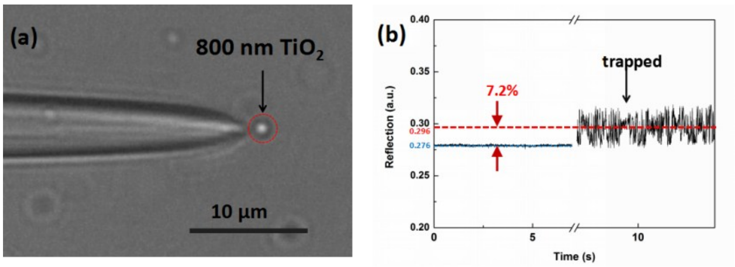


**Fig. S7 (a)** The optical microscope image of trapping 800 nm $TiO_2$ particle. **(b)** Real-time capture of reflection signal for the trapped 800 nm $TiO_2$ particle.

## 8. The optical microscope images and reflection signals of trapping 600, 700, 800 nm *S. aureus*.

Fig. S8 shows the optical microscope images (a–c) and real-time capture signals (d–f) of trapped *S. aureus* with different diameters. The step changes of the 600, 700, 800 nm *S. aureus* are 4.3%, 6.0%, 8.1%, respectively. The amplitude of the step changes of signal increases with the increasing diameter of the *S. aureus*, which is consistent with the conclusions above.

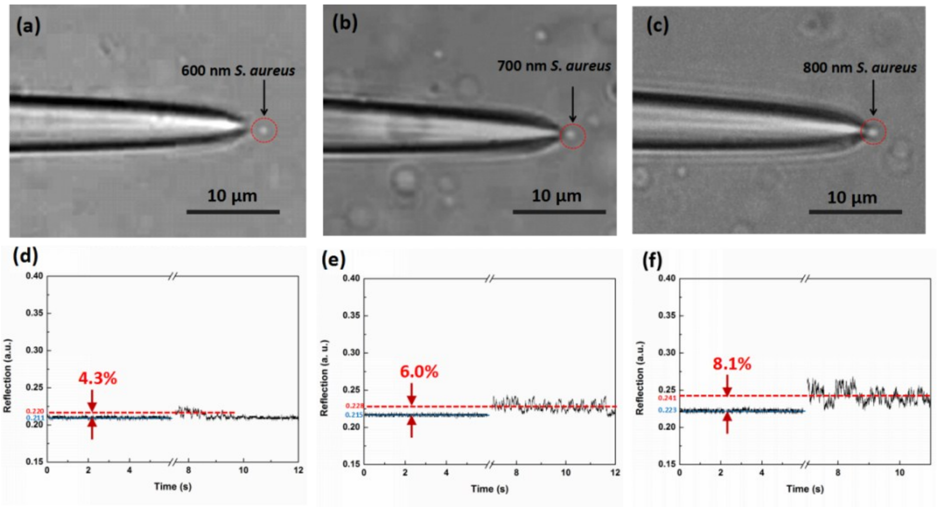


**Fig. S8 (a–c)** The optical microscope images of trapping *S. aureus* with diameters of 600, 700, 800 nm, respectively. **(d–f)** Real-time capture of reflection signals for the trapped *S. aureus* with diameter of 600, 700, 800 nm, respectively.
